# Identifying genetic variants for age of migraine onset in a Han Chinese population in Taiwan

**DOI:** 10.1186/s10194-021-01301-y

**Published:** 2021-08-11

**Authors:** Chia-Kuang Tsai, Chih-Sung Liang, Guan-Yu Lin, Chia-Lin Tsai, Jiunn-Tay Lee, Yueh-Feng Sung, Yu-Kai Lin, Kuo-Sheng Hung, Wei-Liang Chen, Fu-Chi Yang

**Affiliations:** 1 Department of Neurology, Tri-Service General Hospital, National Defense Medical Center, No. 325, Section 2, Cheng-Kung Road, Neihu 114, Taipei, Taiwan; 2 Department of Psychiatry, Beitou Branch, Tri-Service General Hospital, National Defense Medical Center, Taipei, Taiwan; 3 Department of Neurology, Songshan Branch, Tri-Service General Hospital, National Defense Medical Center, Taipei, Taiwan; 4 Center for Precision Medicine and Genomics, Tri-Service General Hospital, National Defense Medical Center, Taipei, Taiwan; 5 Division of Family Medicine, Department of Family and Community Medicine, Tri-Service General Hospital, National Defense Medical Center, Taipei, Taiwan; 6 Division of Geriatric Medicine, Department of Family and Community Medicine, School of Medicine, Tri-Service General Hospital, National Defense Medical Center, Taipei, Taiwan

**Keywords:** Migraine, Age of onset, GWAS, SNP

## Abstract

**Background:**

Considering the involvement of genetics in migraine pathogenesis in diverse ethnic populations, genome-wide association studies (GWAS) are being conducted to identify migraine-susceptibility genes. However, limited surveys have focused on the onset age of migraine (AoM) in Asians. Therefore, in this study, we aimed to identify the susceptibility loci of migraine considering the AoM in an Asian population.

**Methods:**

We conducted a GWAS in 715 patients with migraine of Han Chinese ethnicity, residing in Taiwan, to identify the susceptibility genes associated with AoM. Based on our standard demographic questionnaire, the population was grouped into different subsets. Single-nucleotide polymorphism (SNP) associations were examined using PLINK in different AoM onset groups.

**Results:**

We discovered eight novel susceptibility loci correlated with AoM that reached the GWAS significance level in the Han Chinese population. First, rs146094041 in *ESRRG* was associated with AoM $\le$ 12 years. The other SNPs including rs77630941 in *CUX1*, rs146778855 in *CDH18*, rs117608715 in *NOL3*, rs150592309 in *PRAP1*, and rs181024055 in *NRAP* were associated with the later AoM.

**Conclusions:**

To our knowledge, this is the first GWAS to investigate the AoM in an Asian Han Chinese population. Our newly discovered susceptibility genes may have prospective associations with migraine pathogenesis.

**Supplementary Information:**

The online version contains supplementary material available at 10.1186/s10194-021-01301-y.

## Background

Migraine is the most common debilitating neurological disorder, presenting repeated moderate to severe, throbbing, and unilateral headaches, which may also be accompanied by nausea, vomiting, photophobia, or phonophobia [1, 2]. Approximately 30 % migraineurs have aura characterized by transient neurological symptoms that last for 5–60 min during attacks [3]. Visual symptoms account for more than 90 % cases of migraine with aura [3].

The estimated life-time prevalence of migraine is 15–20 % [1]. Peng et al. reported a prevalence of 9.1 % in Asians [4]. According to the Global Burden of Disease Study (2016), migraine is the second main reason of disability worldwide [2]. These data demonstrate that migraine exerts a significant socioeconomic influence [1]. In clinical practice, the International Classification of Headache Disorders (ICHD) categorizes migraine as migraine with or without aura [3]. The proportion of migraine patients with aura varies between Western and Asian countries. For example, approximately 30 % migraine cases in Western-countries have aura, whereas only 10 % of cases in Asia have aura [4]. Moreover, Asian patients with migraine also have less photophobia [5]. These differences suggest that the pathogenesis of migraine is associated with genetic involvement across diverse ethnic populations.

The prevalence rate of migraine is 4–11 % in children aged 7–11 years in the United States [6]. Early identification of childhood migraine is important because it might disturb daily activities and social functioning, without proper intervention [7–9]. However, the migraine symptoms in this group are difficult to evaluate because children may not be able to express their problem correctly.

Previous studies have shown aggregation in families as a feature of migraine, highlighting its causal genetic architecture. Gormley et al. applied migraine polygenic risk scores (PRS) to investigate the common genetic variant load in 8,319 participants from 1,589 families in Finland [10]. The results of this study indicate that the polygenic load is associated with a lower age-at-onset and severity of migraine [10]. Pelzer et al. revealed that a family history of migraine is associated with an earlier age of onset and increased duration of medication usage in the Netherlands [11]. There are several genetic studies on pediatric migraine. Szilagyi et al. reported that STin2, a polymorphism of the serotonin transporter gene, correlates with pediatric migraine with aura in Hungary, and that it presents with severe abdominal pain and vomiting during the attack [12]. Chang et al. reported that common variants at 5q33.1 correlate with migraine in African–American children [13]. Koute et al. reported that the existence of BDNF rs6265 correlates with a lower risk of pediatric headache and migraine in Greece [14]. However, limited studies have examined the correlation of genetic susceptibility markers and age-at-onset of migraine in an Asian population.

Therefore, the aim of this study was to identify the susceptibility loci of migraine considering the age of migraine onset (AoM) in an Asian population. We hypothesized that some genetic factors may be associated with the AoM. This cohort study involved two-stage clinic-recruitment of migraine participants. We stratified the patients by age groups ≤ 12 years old versus > 12 years old and established a linear regression model for AoM in all cohorts. We aimed to discover susceptibility single-nucleotide polymorphisms (SNPs) associated with the AoM with and without aura in a Han Chinese population residing in Taiwan.

## Methods

### Participants

**Fig. 1:**
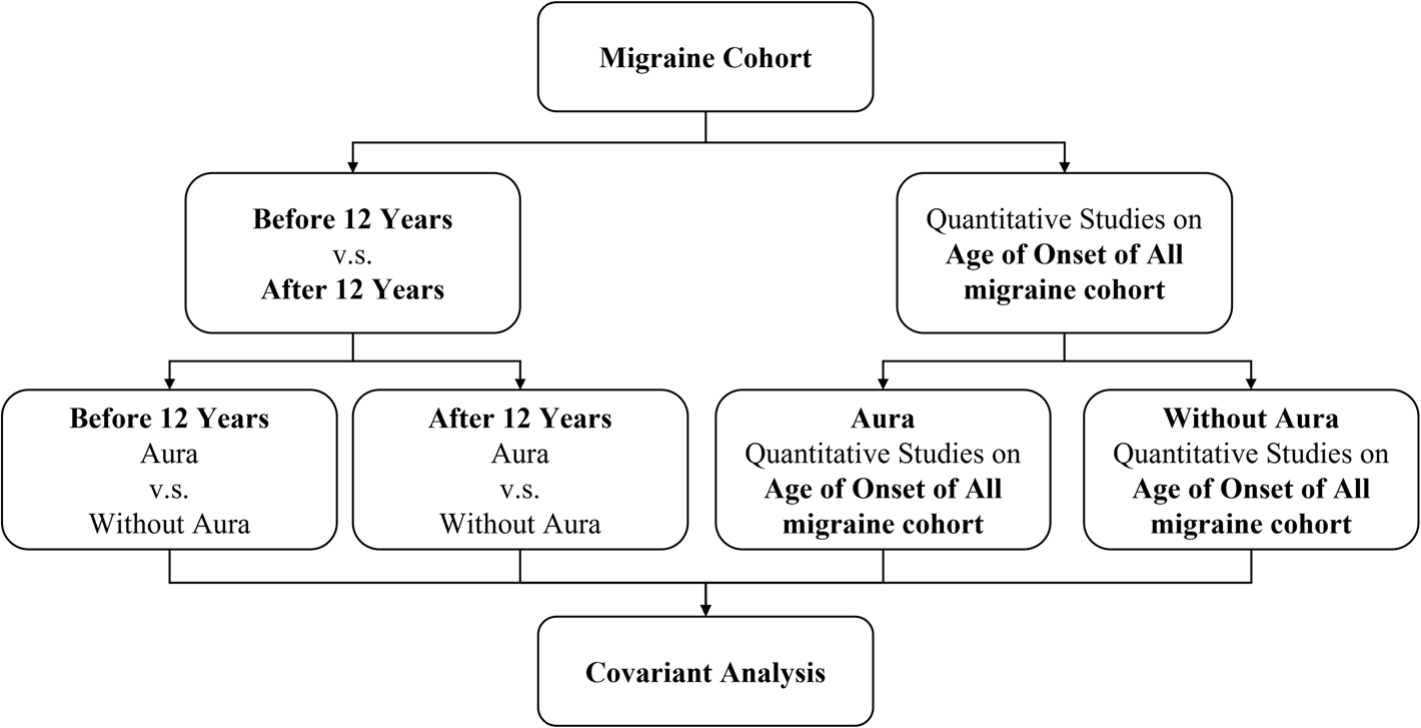
Flowchart showing the pipeline of the variant-phenotype association study. We determined the genotypes and phenotypes of patients with migraine using arrays and a questionnaire, respectively, and the patients were divided based on the onset age of migraine (AoM). In the first stratification, we selected 12-year-old as a limit to separate the patients based on their onset age of migraine into before and after 12-year-old groups in a phenotype association study. We also applied all the migraine cohorts to a quantitative trait association study. The results are shown in Table 2; Fig. 2. In the second stratification, we separated the samples from two studies into two groups based on the presence and absence of aura. The results of the quantitative trait association study in the second stratification are shown in Table 3; Fig. 3

This study involved a cohort of 715 cases of migraine with and without aura. The study protocol was approved by the Tri-Service General Hospital (TSGH) Institutional Review Board. All individuals provided signed, informed consent before enrollment. Each participant completed a screening questionnaire and was later interviewed by a board-verified neurologist and headache consultant (FCY). The diagnosis of migraine was in accordance with the criteria in the third edition of the International Classification of Headache Disorders (ICHD-III) [3]. The participants were then analyzed according to the AoM as shown in Fig. 1. Because the aim of this study was to determine the susceptibility loci associated with AoM, we did not recruit normal controls for comparison in this study.

### Participant evaluation

#### Assessment of migraine

All participants completed a uniform questionnaire, encompassing demographic characteristics, AoM, Migraine Disability Assessment (MIDAS) [15, 16], family history of migraine, body mass index, and years of education. The MIDAS was developed to evaluate headache-associated disability in the past 3 months using a five-item questionnaire. The range of the MIDAS score is 0–270 [15, 16].

The prevalence of migraine is similar between boys and girls in the prepubertal stage [17]. However, the prevalence significantly increases in girls from puberty, around 13–15 years of age. Thus, the cut-off age of pediatric migraine was set as ≤ 12 years in this study to avoid sex hormonal effect [18].

#### Genotyping and quality control

Blood samples from patients were collected in 5-ml EDTA vacutainers (BD, Plymouth, UK). Genomic DNA was extracted from the blood samples using the QIAamp DSP DNA Mini Kit on the QIAsymphony platform (Qiagen, Hilden, Germany). DNA quality was measured using a NanoDrop One spectrophotometer (Thermo Fisher Scientific, Waltham, MA, USA). The genomic DNA was then applied to Affymetrix Axiom Genome-Wide TWB 2.0 arrays, which included approximately 752,921 probes for 686,463 SNPs [19]. The complete list of variants on the TWBv2 array is available at https://www.twbiobank.org.tw/new_web/exp_doc/TWBv2.0_SNPs%E4 %BD%8D%E9 %BB%9E%E7 %9B%B8 %E9 %97 %9 C%E8 %B3 %87 %E8 %A8 %8 A.zip. Of these SNPs, approximately 446,000 SNPs were associated with the Taiwanese genotypic background, 105,000 SNPs were clinically significant, and others are associated with disease, drug response, and metabolism, as determined by Thermo Fisher Scientific over the years. The signal CEL files generated from the Axiom TWB 2.0 SNP array were transformed to genotyping data (tped and tfam) using Analysis Power Tools.

Quality control procedures were conducted using TWB2 SNP criteria and PLINK. All data of DNA quality were tested and recorded, such as OD260/230 > 1.0 and OD260/280 > 1.8, and the concentration > 15 ng/µl. Before genotyping, we eliminated the missing metadata from typed files by clinical metadata for phenotype association study. After gathering all detected loci in arrays, we evaluated the quality of genotyping based on dish quality control (DQC) value > 0.82, average call rate for passing samples > 98.5 %, average genotype calling rates < 97 %, and the Hardy-Weinberg equilibrium (*P* < 0.00001). The platform of genotyping in NCGM is tested annually with thousands of samples to confirm the accuracy of genotyping to correct the standard criteria for all genotyping experiments. Detailed information of Taiwan Biobank is presented on its official website (https://taiwanview.twbiobank.org.tw/index).

### Statistical analysis

Based on our standard demographic questionnaire, the population was grouped into different subsets. SNP associations were examined using PLINK based on the groups for AoM. For the first stratification, in the phenotype association study, we examined the variant relationship between groups with the AoM onset before and after 12 years using the 1df chi-square allelic test, and another quantitative trait association study was applied to determine two different distributions in all migraine cohorts using regression statistics and the Wald test. In the second stratification, all original groups were separated based on the presence and absence of aura and were tested using the quantitative trait association study. Finally, the significant variants were retrieved with *P* *<* 5 E-08 (with *) and were annotated using the NCBI based on the RefSeq database [20] with ANNOVAR [21]. To eliminate common variants in Taiwanese, we filtered candidate variants by MAF (TWB) < 0.25. By further analysis, we collected all genes of candidate variants to perform functional annotation clustering using DAVID Bioinformatics Resources 6.8 [22]. In addition, we mapped all candidate variants to the GTEx v8 project (https://gtexprotal.org) by downloading 50 tissue cis-expression quantitative trait locus (cis-eQTL) data with FDR cut-off 5 % to identify the relationship of variant-gene regulation [23].

## Results

### Demographics

Table 1 shows the demographic metadata of participants according to the migraine type, that is, with and without aura. There were no significant differences in sex, migraine frequency, family history, or body mass index among the participants. However, patients with migraine were significantly different in terms of age, age of onset, and the MIDAS score (*P* < 0.05). Migraineurs with aura had earlier AoM and higher MIDAS scores than migraineurs without aura.

**Table 1:** Demographic and clinical data

	All-Migraine	All-Migraine		***P***-value
		Aura	Without Aura	
**Migraine cohort**	715	167	548	NA
**Age, years**	43.65 ± 12.34	39.51 ± 12.70	45.53 ± 11.72	5.91 × 10^-7^
**Female**	565 (79.0)	129 (77.2)	436 (79.5)	0.64
**AoM, years**	21.20 ± 9.24	18.90 ± 8.38	22.24 ± 9.43	2.0 × 10^-4^
**Migraine frequency**	7.44 ± 7.11	8.35 ± 7.06	7.04 ± 7.10	0.051
**MIDAS score**	19.94 ± 16.74	23.85 ± 17.16	18.18 ± 16.27	4.23 × 10^-4^
**Family history of migraine**	347 (48.5)	92 (55.0)	255 (46.5)	0.64
**Body mass index**	23.40 ± 4.02	23.72 ± 3.72	23.26 ± 4.10	0.21
**Education, years**	14.01 ± 2.96	14.17 ± 2.96	13.94 ± 2.96	0.40

*P*-value was determined using Fisher’s exact test and the *t*-test

*AoM* age of onset of migraine, *MIDAS* migraine disability assessment scale, *NA* not available

### Associations with AoM Onset

**Fig. 2:**
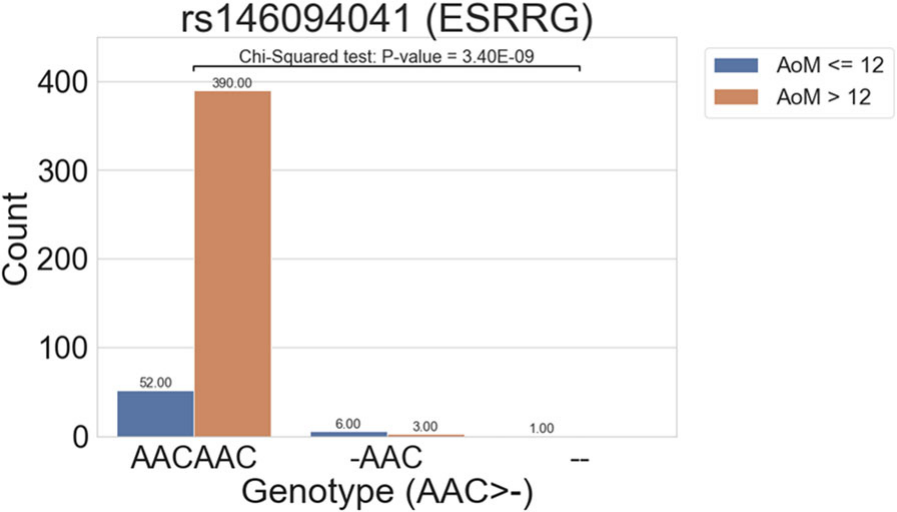
Boxplots of distributions between the groups and genotypes. The x-axis shows the genotype of the variant and the y-axis shows the phenotype. The abundance of each condition in the genotypes is marked above each bar. We found three different binary distributions on the variant rs146094041. The genotype CAA (wild type, WT) is associated with the control groups (after 12 years), whereas the variants with one allele or double deletion are associated with the case groups (before 12 years). ESRRG, estrogen-related receptor gamma

The migraine cohort was separated based on AoM onset into before and after 12-year-old groups (Fig. 1). In the association analysis, we found that one deletion, rs146094041 in *ESRRG* (*P* = 3.40 E-09), was more significant, with *P* < 5 E-8 (Table 2). The deletion allele frequency for rs146094041 in *ESRRG* was 0.38 % in the before 12-year-old group and 6.78 % in the after 12-year-old group (odds ratios 0.053, $\le$12 group as reference). More deletion alleles were observed in the before 12-year-old group (Fig. 2A), whereas the wild type allele (AAC) was more in the after 12-year-old group.

**Table 2:** Associations with onset age of migraine

								Risk allele frequency			
**Age of Onset**	**SNP**	**Position (GRCh38.p12)**	**MAF**	**TWB**	**Gene**	**Type**	**Variant Change**	**≤ 12**	**> 12**	***P*-value**	**OR**
≤ 12 vs. >12	rs146094041	chr1:217,092,941	0.01	0.02	ESRRG	intronic/Del	CAA > -	6.78 %	0.38 %	3.40 × 10^− 9*^	0.053 [0.014,0.20]

Patients with migraine were grouped based on the age of onset and were compared using PLINK

*ESRRG* estrogen-related receptor gamma, *MAF* minor allele frequency of East Asian group in dbSNP, *TWB* minor allele frequency in the Taiwan Biobank

Significant variants with *P* <  5× 10^− 8^ are listed with the allele frequency and odds ratio (OR) with 95 % confident interval for the phenotype association study on the onset of migraine before or after 12 years of age

* is the criteria whose *P*-value was <$5\times {10}^{-8}$

### Associations in the Subgroups of the < 12-Year-Old Group

We separated both onset age-based groups into subgroups based on the presence and absence of aura (Fig. 1). However, no variant reached the threshold *P* < 5 E-8 in the analysis of both subgroups.

### Associations in All Migraine Cohorts of AoM

**Fig. 3:**
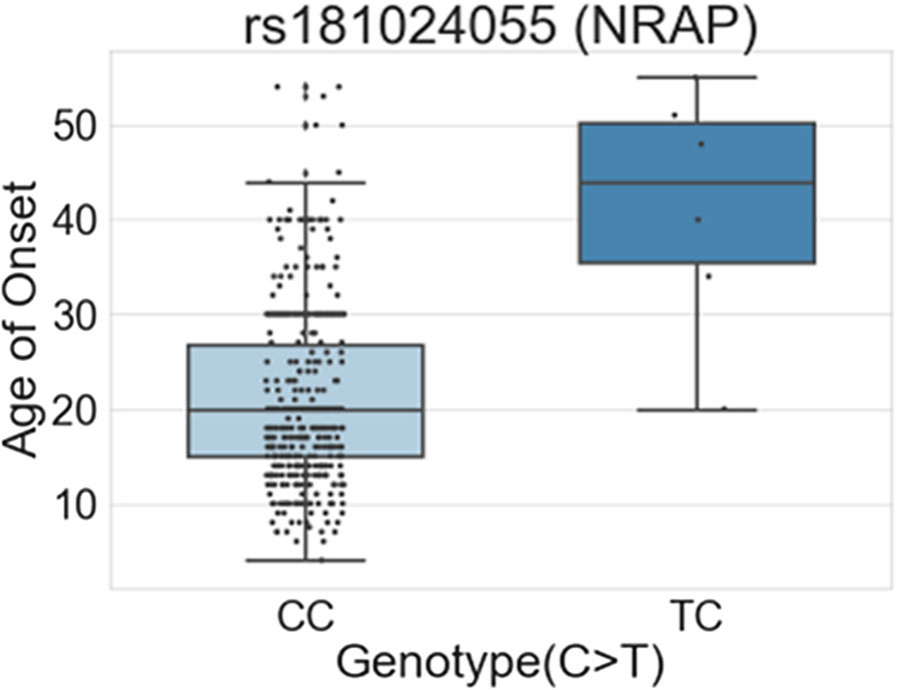
Boxplots of quantitative distributions between genotypes and subgroups in the all migraine cohort. The x-axis shows the genotype of the variant and the y-axis shows the quantitative phenotype (age). NRAP, nebulin-related anchoring protein

We also performed a quantitative trait association study on all migraine cohorts (Fig. 1). The results showed that SNP rs181024055 in the *NRAP* exonic region (*P* = 4.887E-08) has a more significant value than *P* < 5 E-8 in two different AoM distributions (Table 3; Fig. 3). The AoM median with genotype TC (approximately AoM = 44 years) was higher than that with the wild genotype CC (AoM = 20 years, β value of regression coefficient = 20.41). According to the dbSNP and Taiwan Biobank database, the frequency of the T allele was 0.022 (4,500 samples) in the East Asian population and 0.003 in Taiwanese population, respectively. Moreover, patients with genotype TC were likely to have higher age-at-onset migraine than patients with the wild type.

**Table 3:** Association of onset age of the all-migraine cohort in the subgroups with or without aura

Group	SNP	Position (GRCh38.p12)	MAF	TWB	Gene	Type	Variant Change	***P***-value	***β*** (Beta)
All migraine cohort	rs181024055	chr10:113595726	0.022	0.003	NRAP	exonic	C > T	4.89 × 10^-8*^	20.41
Aura	rs77630941	chr7:102217020	0.020	0.032	CUX1	intronic	T > C	6.66 × 10^-10*^	28.37
	rs146778855	chr5:20412078	0.020	0.014	CDH18	intronic	C > T	1.21 × 10^-8*^	20.63
	rs117608715	chr16:67172270	0.010	0.030	NOL3	intronic	C > T	1.78 × 10^-8*^	22.74
	rs150592309	chr10:133346685	0.00	0.024	PRAP1	upstream	G > A	8.88 × 10^-8^	21.72
Without aura	rs181024055	chr10:113595726	0.022	0.003	NRAP	exonic	C > T	4.89 × 10^-8*^	23.74

A quantitative association study was performed on the all-migraine cohort of AoM and was, then, grouped based on the presence and absence of aura. Significant variants with *P* < 5 × 10^-8^ are listed with the beta value of the regression coefficient

*CUX1* cut like homeobox 1, *CDH18* cadherin 18, *NOL3* nucleolar protein 3, *PRAP1* proline rich acidic protein 1, *NRAP* nebulin related anchoring protein; minor allele frequency of the East Asian group in dbSNP, *TWB* Minor allele frequency in the Taiwan Biobank

^*^is the criteria whose *P*-value < 5 × 10^−8^

### Associations in the subgroups with aura

**Fig. 4:**
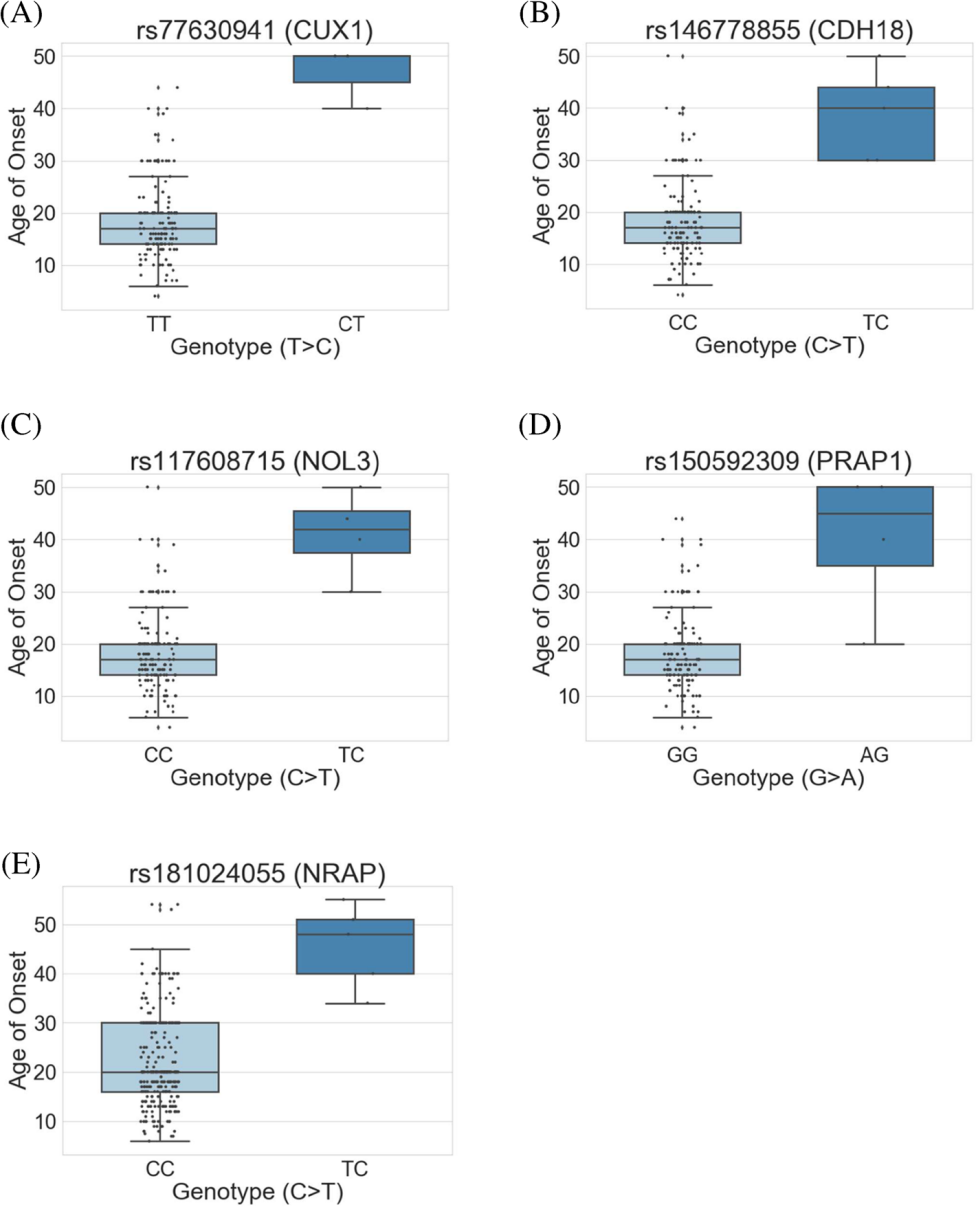
Boxplots of quantitative distributions between genotypes and onset age of migraine subgroup with (**a-d**) and without aura (**e**). The x-axis shows the genotype of the variant and the y-axis shows the quantitative phenotype (age). CUX1, cut like homeobox 1; CDH18, cadherin 18; NOL3, nucleolar protein 3; PRAP1, proline rich acidic protein 1; NRAP, nebulin-related anchoring protein

In all migraine cohorts with aura, 3 SNPs had *P* < 5 E-08 (rs77630941 in *CUX1*, rs146778855 in *CDH18*, and rs117608715 in *NOL3*) and 1 SNP (rs150592309 in *PRAP1*) were almost significant in the aura groups (*P* = 6.66 E-10, *P* = 1.21 E-08, *P* = 1.78 E-08, *P* = 8.88 E-08, respectively in Fig. 4A–D). Three SNPs belonged to intron regions and rs150592309 was in an upstream region (Table 3). Patients with alternative genotypes likely had later onset migraine than those with the wild type (β = 28.37, β = 20.63, β = 22.74, β = 21.72, respectively).

### Associations in the subgroups without aura

We also compared the migraine groups without aura. In the before and after 12-year-old groups, no variants reached the threshold (*P* < 5 E-08). However, we found that the exonic variant rs181024055 in *NRAP* (*P* = 4.89 E-08, β = 23.74) significantly reached the threshold (Table 3; Fig. 4E). Interestingly, the variant was the same as in the previous finding in all migraine cohorts (Table 3). For rs181024055 in *NRAP*, the median AoM in genotype CC (AoM = 20 years) was lower than that in the alternative genotype TC (approximately AoM = 48 years). We thus suggest that this variant is more associated in the cohorts of migraine without aura.

## Discussion

To our knowledge, this is the first genome-wide association study to report eight SNPs associated with AoM in an Asian population with migraine. Evaluating the genetic susceptibility associated with the (endo)phenotypes and characteristics of migraine facilitate diagnosis, identification of pathology, and eventual discovery of targets for therapy. In this study, rs146094041 in *ESRRG* and rs7124169 in chromosome 11 were found to be more susceptible to early AoM with ≤ 12 years. SNP rs181024055 in *NRAP* was associated with later AoM in all migraine cohort analysis. Furthermore, 4 SNPs, rs77630941 in *CUX1*, rs146778855 in *CDH18*, rs117608715 in *NOL3*, and rs150592309 near *PRAP1*, were found to be correlated with late AoM in the aura group. Furthermore, rs181024055 in *NRAP* is associated with late AoM in the migraine without aura group.

*ESRRG* encodes estrogen-related receptor gamma (ERRγ), also known as nuclear receptor subfamily 3, group B, member 3 (NR3B3). ERRγ is an orphan receptor to a member of nuclear steroid hormone receptors. Estrogen signaling and estrogen-related receptors have been extensively associated with the pathogenesis of auditory function and migraine [24–26]. For example, Nolan et al. demonstrated that *ESRRG* expression is highly upregulated in cochlear hair cells and that it is linked with a candidate gene for senile hearing impairment [27]. Schilit et al. reported that the disruption of *ESRRG* on chromosome 1 is involved in a phenotype including hearing impairment and delayed development [28]. Moreover, a nationwide population-based survey revealed that migraineurs have a higher risk of acquiring sudden sensorineural hearing loss than the control individuals [29]. Additionally, ERRγ and nociception-related genes have been reported as targets of bisphenol A, a potential contributor that aggravates migraine-like behaviors in a rat model, which may link its association with migraine [30]. These findings provide a potential linkage between *ESRRG* and the mechanism of auditory symptoms as well as the environmental effects on migraine.

*NRAP* encodes nebulin-related anchoring protein, a muscle-specific isoform belonging to the nebulin protein family. NRAP is associated with the link between mature muscle cells and myofibrillar myogenesis during growth. *NRAP* mutation is associated with dilated cardiomyopathy [31, 32]. The involvement of *NRAP* in migraine pathogenesis has not been described. Thus, NRAP may have a novel role in migraine pathogenesis.

*CUX1* encodes cut like homeobox 1 (Cux1), a homeobox transcription factor that is expressed ubiquitously in all mammalian tissues. Cux1 is involved in regulating gene expression, cell cycle progression, morphogenesis, and differentiation [33]. In the nervous system, Cux1 is recognized as a specific molecular marker for the pyramidal neurons in the upper layers (II-IV) and is expressed during neurogenesis [34]. Li et al. reported that Cux1 acts as a downregulator of dendritic development for cortical pyramidal neurons by repressing the expression of the cyclin-dependent kinase inhibitor, p27^Kip1^ [24]. Cubelos et al. demonstrated that Cux1-knockout mice have impaired working memory due to abnormal dendritogenesis and decreased synaptic function [35]. Furthermore, in a genome-wide association study in an East Asian population, Liu et al. found that Cux1 is associated with autism spectrum disorders [36]. Overall, these findings support that *CUX1* is functionally involved in migraine.

*CDH18* encodes cadherin 18, a type II classical cadherin that mediates calcium-dependent cell-cell adhesion. *CDH18* is expressed particularly in the brain and is involved in regulating neural morphogenesis, including the formation and plasticity of synapses [37]. Structural variations of *CDH18* are associated with autism spectrum disorders [38]. Additionally, Terracciano et al. reported that SNPs near the *CDH18* gene show a robust association with depression [39]. Overall, these studies support the potential effects of *CDH18* on the mechanisms involved in migraine.

*NOL3* encodes nucleolar protein 3 (NOL3) in humans. NOL3 is highly expressed in several organs including the skeletal muscle, heart, and the brain. NOL3 has a functional CARD domain that acts as an apoptosis repressor by downregulating caspase-2 and caspase − 8 activities [40, 41]. Jonathan et al. reported that familial cortical myoclonus is caused by a mutation in *NOL3*. Further *in vitro* and *in vivo* experiments verified that *NOL3* mutation alters the post-translational modification of NOL3 and that neuronal hyperexcitability is absent in *Nol3* knockout mice [24]. *NOL3* mutation may also cause delayed AoM by a loss or decrease-of-function in neuronal excitability, although the exact pathogenesis associating *NOL3* and migraine needs further investigation.

This study has several strengths. First, all participants underwent a structured interview through authenticated questionnaires. Thus, we cautiously excluded cases of other types of headaches, particularly tension type and medication overuse headaches, which could alter the clinical features. Second, the genes associated with AoM onset in this study are mostly expressed in the nervous system and are involved in neuronal excitability and neurogenesis. Third, migraine is associated with a high prevalence of psychiatric comorbidities including mood and autism spectral disorders. The potential mechanism of migraine comorbid mood disorders includes the dysregulation of the normal sensory network and alternation of functional connections between the brainstem-regulating circuits and the neuro-limbic centers [42, 43]. Neuro-imaging surveys provide evidence that psychiatric disorders and migraine have a common matrix because they share dysfunctional pain-related network areas such as the amygdala and periaqueductal gray matter [43, 44]. As the AoM genes reported in this study are mostly known to be involved in psychiatric disorders, further in-depth investigation regarding the association between psychiatric disorders and AoM-related genes could provide a better understanding of how these factors might influence the migraine/mood relationship.

This study also has some limitations. First, the sample size of this study is relatively small and may partially limit its statistical power. Enrolling more migraineurs, especially aura participants, is thus warranted to validate these results in the future. Second, the proportion of patients in the aura group in this study, 23.3 %, is higher than that in a previous Asian study (10 %). This could be because the participants were all recruited from Dr. Yang’s outpatient clinic focusing on headache. Dr. Yang is a headache specialist and he received referral of patients with intractable headache from clinics, especial migraine with aura because of its unique presentation and higher risk of comorbidity [45]. This is evidenced by the higher MIDAS score of the aura group than that of the non-aura group in Table 1. Thus, there was a higher proportion of aura patients in our migraine cohort. Third, we did not recruit normal control group in the current survey because the study goal was to identify SNPs associated with AoM in migraineurs. Thus, we checked the allele frequency of these AoM-associated SNPs in Taiwan Biobank, a well-known database that collects information of healthy Taiwanese population [19, 46]. The results confirmed that these significant variants were not common variants (MAF < 5 %). Fourth, the correlation of mechanism and functional analysis of these implicated genes and migraine is limited in the current study. We tried functional clustering of these AoM-related genes using DAVID Bioinformatics Resources 6.8 [22]. As shown in Supplementary Table 1, the results revealed that the genes of variants were associated with splice variant category without statistical significance. To determine the trend of variant-gene association in human tissues, we also mapped the variants to the GTEx v8 database [23]. However, the variants were not mapped to any tissue because the ethnic groups in the GTEx project did not include Taiwanese. Further research on the functional effects of these SNPs is required to better recognize their correlation with the onset of migraine and to elucidate the precise molecular mechanisms. Finally, these AoM-related SNPs are novel candidates and none of them have been reported in previous migraine-related GWAS. The comparison and validation of the results in an independent population are needed. Besides, we tried to replicate previously reported variants contributing to migraine in our cohort [10, 12–14, 47]. However, all these variants were not included in the TWB array, because it was designed based on genotyping data of Taiwanese Han Chinese participants [19]. In the past 20 years, various studies have reported candidate genes involved in the pathogenesis of migraine or other disease, but the replication and concordance in another ethnic populations are often unsatisfactory [48, 49]. Although genetic factors are affected by ethnicities, the discovery of different loci will help to identify undiscovered characteristics of migraine pathophysiology, which may assist in the development of new treatment [7].

## Conclusions

Several novel susceptibility genes associated with AoM, most of which were probably insinuated in migraine, were identified in this study. Thus, this study will enhance the diagnosis and treatment of patients with migraine; however, a larger sample is necessary to obtain the definitive proof.

## Supplementary Information

**Additional file 1: Supplementary Table 1.** Functional Clustering Analysis of genes of candidate variants

## Data Availability

All data are available from the corresponding author upon request.

## Abbreviations

AoM: Age of onset of migraine
BPD: Borderline personality disorder
CDH18: Cadherin 18
CUX1: Cut like homeobox 1
ESRRG: Estrogen related receptor gamma
GWAS: Genome-wide association studies
ICHD: International Classification of Headache Disorders
MAF: Minor allele Frequency of East Asian group in dbSNP
MDD: Major depressive disorder
MIDAS: Migraine Disability Assessment
NA: Not available
NDSP: Neuroblastoma-derived secretory protein
NOL3: Nucleolar protein 3
NR3B3: Nuclear receptor subfamily 3, group B, member 3
NRAP: Nebulin related anchoring protein
OR: Odds ratio
PRAP1: Proline rich acidic protein 1
SNP: Single-nucleotide polymorphism
TWB: Minor allele frequency in Taiwan Biobank
WT: Wild type
