# Supplementary material for: Identifying genetic variants for age of migraine onset in a Han Chinese population in Taiwan
Source: J Headache Pain. 2021 Aug 11;22(1):89. doi: 10.1186/s10194-021-01301-y (PMC8356430; doi:10.1186/s10194-021-01301-y)
Supplement: Supplementary file 1 — Additional file 1: Supplementary Table 1. Functional Clustering Analysis of genes of candidate variants [file 10194_2021_1301_MOESM1_ESM.docx]

**Supplementary Table 1** Functional Clustering Analysis of genes of candidate variants

| **Category** | | **Term** | **Count** | **%** | **P-Value** | **Genes** | **Fold Enrichment** |
| --- | --- | --- | --- | --- | --- | --- | --- |
| UP_SEQ_FEATURE | splice variant | | 5 | 83.33 | 0.077 | CUX1, NRAP, ESRRG, NOL3, PRAP1 | 2.154532 |
| UP_KEYWORDS | Polymorphism | | 4 | 66.67 | 0.66 | CUX1, NRAP, ESRRG, PRAP1 | 1.139306 |
| UP_SEQ_FEATURE | sequence variant | | 4 | 66.67 | 0.72 | CUX1, NRAP, ESRRG, PRAP1 | 1.074928 |
